# Oral mucositis and selective elimination of oral flora in head and neck cancer patients receiving radiotherapy: a double-blind randomised clinical trial

**DOI:** 10.1038/sj.bjc.6600824

**Published:** 2003-04-01

**Authors:** M A Stokman, F K L Spijkervet, F R Burlage, P U Dijkstra, W L Manson, E G E de Vries, J L N Roodenburg

**Affiliations:** 1 Department of Oral and Maxillofacial Surgery, University Hospital Groningen, P.O. Box 30.001, 9700 RB Groningen, The Netherlands; 2 Department of Radiotherapy, University Hospital Groningen, The Netherlands; 3 Department of Microbiology, University Hospital Groningen, The Netherlands; 4 Department of Medical Oncology, University Hospital Groningen, The Netherlands

**Keywords:** mucositis, head and neck cancer, antibiotics, radiotherapy

## Abstract

Mucositis is an acute inflammation of the oral mucosa because of radiotherapy and/or chemotherapy. All patients receiving radiotherapy in the head and neck region develop oral mucositis. The aim of this study was to analyse the effects of selective oral flora elimination on radiotherapy-induced oral mucositis, in a double-blind, randomised, placebo-controlled trial. Sixty-five patients with a malignant tumour in the head and neck regions to be treated with primary curative or postoperative radiotherapy participated in this study. The patients received either the active lozenges of 1 g containing polymyxin E 2 mg, tobramycin 1.8 mg and amphotericin B 10 mg (PTA) (33 patients) or the placebo lozenges (32 patients), four times daily during the full course of radiotherapy. Mucositis, changes in the oral flora, quality of feeding and changes of total body weight were assessed. Mucositis score did not differ between the groups during the first 5 weeks of radiotherapy. Nasogastric tube feeding was needed in six patients (19%) of the placebo group and two patients (6%) of the PTA group (*P*=0.08). Mean weight loss after 5 weeks of radiation was less in the PTA group (1.3 kg) (s.d.: 3.0) than in the placebo group (2.8 kg) (s.d.: 2.9) (*P*=0.05). Colonisation index of *Candida* species and Gram-negative bacilli was reduced in the PTA group and not in the placebo group (*P*<0.05). No effect on other microorganisms was detected. In conclusion, selective oral flora elimination in head and neck irradiation patients does not prevent the development of severe mucositis.

Radiotherapy in head and neck cancer patients can induce oral mucositis, which is an acute inflammation of the oral mucosa. Until now no effective intervention has been developed to prevent oral mucositis in radiotherapy (Sutherland and Browman, 2001). This prevention is even more relevant now because altered fractionation schedules for the treatment of head and neck malignancies induce more severe mucositis (Kaanders *et al*, 1999). All patients receiving radiotherapy in the head and neck region develop oral mucositis to some extent, depending on radiation schedule, radiation field, radiation volume and cumulative dose (Miralbell *et al*, 1999). Clinically, mucositis appears in a conventional radiation scheme after a cumulative radiation dose of 10–20 Gy as a white discoloration of the mucosa because of hyperkeratinisation. The next stage is a deepening erythema followed by the development of pseudomembranes and ulcerations. Severe mucositis, appearing as pseudomembranes, will develop at the end of the third week of radiation, after about 30 Gy (Baker, 1982; Spijkervet *et al*, 1989b). Prevention of severe mucositis is important because mucositis affects the patient's feeding status, physical and mental well-being and it can influence the course of radiotherapy (Sutherland and Browman, 2001). Further oral pain because of mucositis has a serious impact on the quality of life of patients (Miralbell *et al*, 1999).

Several mechanisms are supposed to play a role in the development of mucositis: changes at the cellular level of the basal cell layer, inflammatory process in the epithelium and influence of bacteria on mucosal surface. Changed oral flora, colonising the oral mucosa, may aggravate the mucosa reaction because of radiation (Van Saene and Martin, 1990). The carriage and colonisation of aerobic Gram-negative bacilli are thought to play a role in the pathogenesis of irradiation mucositis (Bernhoft and Skaug, 1985). A hypothesis has been proposed on the development of mucositis in four consecutive phases, in which the ulcerative/bacterial phase is thought to play a role in the development of fibrous pseudomembranes of the oral mucosa (Sonis, 1998). A pilot study in 15 patients reported the protective effect of an antibiotic lozenge for selective elimination of the oral flora (Spijkervet *et al*, 1990). Less severe mucositis and a less mean mucositis score compared to a historical control group was observed. None of the PTA-(polymyxin E 2 mg, tobramycin 1.8 mg and amphotericin B 10 mg) treated patients needed nasogastric tube feeding. In a cohort study including 36 patients, it was found that PTA lozenges may reduce irradiation mucositis (Kaanders *et al*, 1995). In contrast, randomised studies reported conflicting effects on mucositis by selective oral flora elimination (Symonds *et al*, 1996; Okuno *et al*, 1997; Wijers *et al*, 2001).

The aim of this study was to evaluate in a randomised, double-blind, placebo-controlled trial the effects of selective oral flora elimination on the development of irradiation-induced oral mucositis, feeding, weight loss and colonisation of aerobic Gram-negative bacilli and yeast.

## PATIENTS AND METHODS

### Protocol

Patients with a malignant tumour in the head and neck regions to be treated with primary curative or postoperative radiotherapy were eligible for this study. Inclusion criteria for the study were: external bilateral irradiation via parallel-opposed portals by a linear accelerator (4–6 MeV), fractionation of 2 Gy daily, five times a week, with a prescribed dose of at least 50 Gy and at least 50% of the oral mucosa in the field of radiation. The dose specification was in line with ICRU 50 recommendations (Anonymous, 1993).

Criteria for exclusion were: (1) an oral mucosa defect other than related to tumour surgery; (2) need for an obturator or resection prosthesis and; (3) treatment with antibiotics for an oral infection the last 2 weeks before the start of irradiation.

As a standard procedure all patients were evaluated before radiation treatment for potential risk factors for oral complications by means of a thorough oral and dental evaluation, including a radiographic examination. All potential risk factors were eliminated appropriately before the start of radiotherapy. The supportive oral care regimen consisted of a daily protocol of cleansing the oral cavity by means of spraying with saline by the dental hygienist, and mouth rinsing by the patients with a salt–baking soda solution at least eight times a day to remove sticky saliva and debris. Dentate patients applied a neutral fluoride gel every second day with custom-made trays and edentulous patients were not allowed to wear their dentures during the course of radiotherapy (Jansma *et al*, 1992).

The Medical Ethical Committee approved the study and all eligible patients gave written informed consent.

### Assignment

The eligible patients were randomised to receive active lozenges of 1 g containing polymyxin E 2 mg, tobramycin 1.8 mg and amphotericin B 10 mg (PTA) or placebo lozenges. The ingredients of the placebo lozenge were identical with the PTA lozenge except the active drugs. The colour, taste and form of the PTA and placebo lozenges were identical as well. Randomisation was performed by the hospital pharmacist according to a computer-generated, randomised allocation schedule. Patients, clinicians, dental hygienists and microbiologists were blind for who was taking antibiotics. The patients used a PTA or placebo lozenge four times daily starting the first day of irradiation during the total radiation period.

### Assessments

The study period included only the first 5 weeks of radiation because of the wide range of field changes above 50 Gy of radiation. During the study period mucositis, feeding and body weight scores were performed at the start of radiotherapy and twice weekly (Monday–Thursday). The assessments were performed by an assigned dental hygienist. For each patient a mean weekly score was calculated on the basis of these two scores. These mean scores were used for further statistical analyses.

Twice weekly (Monday–Thursday) and two times before the start of radiation oral washings were obtained to examine the oral flora for Gram-negative bacilli, *Candida* species, viridans streptococci, *Enterococci*, *Staphylococcus aureus* and coagulase-negative *Staphylococci*.

#### Mucositis

The mean mucositis was scored by using qualitative and quantitative parameters (Spijkervet *et al*, 1989b). Four different local signs of mucositis (*k*) might be distinguished: 1=white discoloration; 2=erythema; 3=formation of pseudomembranes; 4=ulceration. Mucositis of the oral cavity was determined for maximally eight distinguishable irradiated areas of the mouth: buccal mucosa (left and right), soft and hard palates, dorsum and border of the tongue (left and right), and the floor of the mouth. The degree of mucositis of each area was scored according to the local signs of mucositis. The length (*E*) of the local sign of mucositis was measured: 1=⩽1 cm; 2=1–2 cm; 3=2–4 cm; 4=⩾4 cm. The degree of mucositis was defined as the product of the values *k* and *E*. The mucositis score was defined as the mean of the scores assigned to the irradiated areas.

The mucositis was also scored according to the WHO score (grade 0=normal, no mucositis; grade 1=soreness and erythema; grade 2=erythema, ulcers, can eat solids; grade 3=ulcers, requires liquid diet only; grade 4=alimentation not possible) (Anonymous, 1979).

#### Feeding

The quality of feeding was scored (0=normal, no changes; 1=symptoms without medication; 2=symptoms with medication; 3=liquid diet only; 4=nasogastric tube feeding) and body weight was determined. Afterwards the changes in weight were scored.

#### Microbiological methods

To acquire an oral washing, patients gargled and rinsed their mouth with 10 ml sterile saline for 30 s, and spit it into a sterile vial.

One millilitre of the sample was diluted in 9 ml of Brain Heart infusion (BHI) (Oxoid, Basingstoke, England) and this suspension was serially diluted in BHI. The suspensions were than plated out onto 5% sheep blood agar, McConkey-3 agar (Oxoid) and Yeast morphology agar (Merck, Darmstadt, Germany). The agar plates and BHI broth cultures tubes were incubated overnight aerobically at 37°C. If an agar plate did not show growth and the corresponding BHI broth culture of the dilution series did show turbidity, then this suspension was plated again onto the agars mentioned above. With this enrichment step even low numbers of *Candida* species and Gram-negative bacilli could be detected (Spijkervet *et al*, 1989a). By reading and counting the plates after incubation the viable numbers of microorganisms per millilitre was estimated. The identification was performed by standard microbiological techniques.

#### Definitions

Carriage of a particular microorganism was defined as the condition in which a patient showed a minimum of two consecutive oral washings positive for that organism.

Colonisation index of the oral cavity was defined as the sum of logarithms of the concentrations of a particular microorganism isolated from 1 ml of oral-washing specimens divided by the number of oral washings.

### Statistical analysis

Sample size calculation of this study was based on the study by Spijkervet (Spijkervet *et al*, 1990). A two-sided *α* of 5% and a power of 80% were used. Additionally, a 50% reduction of mucositis in the PTA group was determined as clinically relevant with a normal incidence of mucositis of 80%. Based on these assumptions, 27 patients in each group would be sufficient.

Intention-to-treat analysis was performed. The difference of dropouts between both groups was analysed using Fisher's exact test.

The results were analysed, with respect to mean mucositis, the loss of weight, and colonisation numbers for five different microorganisms (*t*-test for independent samples) and the WHO mucositis score and feeding (Mann–Whitney *U* test). Two-sided tests, performed at the 5% level of significance, were used.

## RESULTS

Patient characteristics are shown in Table 1

**Table 1:** Patient characteristics

**Patients characteristics**	**Placebo**	**PTA**	***P*-value**
aAge mean±sd (years)	54 (10.8)	56 (12.5)	0.36
Gender: male/female (*n*)	22/10	24/9	0.79
			
*Tumor site*			
Oral cavity (*n*)	17	23	0.41
Oropharynx (*n*)	10	8	
Hypopharynx (*n*)	1	1	
Unknown primary(*n*)	4	1	
			
*Histology*			
Squamous (*n*)	31	32	1.0
Other (*n*)	1	1	
			
*T-stage*			
T1 (*n*)	4	5	0.25
T2 (*n*)	6	5	
T3 (*n*)	8	8	
T4 (*n*)	9	14	
bTx (*n*)	5	1	
			
*N-stage*			
N0 (*n*)	10	14	0.12
N1 (*n*)	6	10	
N2a (*n*)	2	1	
N2b (*n*)	9	3	
N2c (*n*)	1	3	
N3 (*n*)	4	2	
			
*Surgery*			
Yes (*n*)	21	27	0.17
No (*n*)	11	6	
			
*Dentures*			
Yes (*n*)	20	25	0.29
No (*n*)	12	8	

All differences between the groups were analysed using *χ*^2^ test except

a in which *t*-test was used.

b Six patients with an unknown primary tumour.

. From January 1994 to February 1997, 65 patients were included, 33 patients received PTA lozenges and 32 the placebo lozenges. Out of the 65 included patients, 58 patients were evaluable for the total evaluation period of 5 weeks. Seven patients (11%) dropped out earlier from the study, five of the PTA group (15%) and two of the placebo group (6%). The difference of dropouts between both groups was not significant. One patient (PTA) developed a skin reaction, unlikely caused by the PTA lozenges, and one patient (placebo) could not suck the lozenges because of the tumour surgery of his tongue. The other five dropped out for reasons not related to one of the lozenges. Of the seven dropouts, one patient stopped after 1 week, two patients after 2 weeks, one patient after 3 weeks and three patients after 4 weeks radiation.

### Mucositis

**Figure 1:**
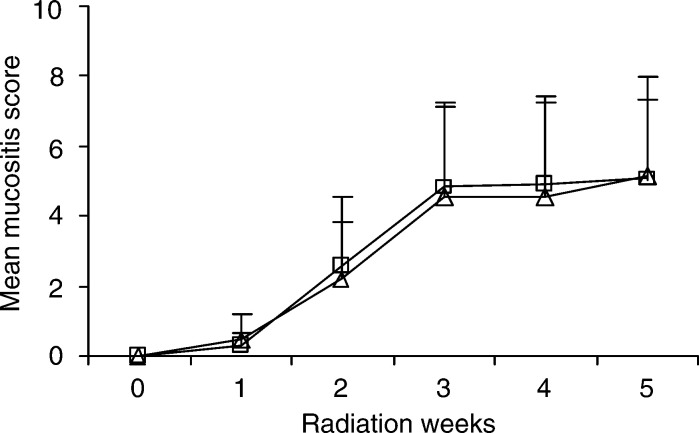
The mean mucositis score (±s.d.) for the PTA group (▵) and the placebo group (□).

The mean mucositis was the same in the PTA group and the placebo group during the study period (*P*>0.2) (Figure 1

). During the 5-week observation period 89% of the patients in the PTA group developed pseudomembranes and in the placebo group 94%. The mucositis according to the WHO score did not differ throughout the study period between both groups (*P*>0.5). In the PTA group, 80% of the patients developed grades 3 and 4 mucositis according to the WHO score, and in the placebo group 90%. The appearance of pseudomembranes was for both groups at a similar radiation status; after 30 Gy of radiation.

### Feeding

Six patients (19%) in the placebo group (*n*=32) and two patients (6%) in the PTA group (*n*=33) needed nasogastric tube feeding during the evaluation period (*P*=0.08).

### Body weight

The mean weight loss after 5 weeks of radiation was less in the PTA group by 1.3 kg (s.d. 3.0) than in the placebo group 2.8 kg (s.d. 2.9) (*P*=0.05).

### Microorganisms

For viridans streptococci, *Enterococci*, *Staphylococcus aureus* and coagulase-negative *Staphylococci*, a similar pattern of carriage and colonisation was found in both groups.

**Figure 2:**
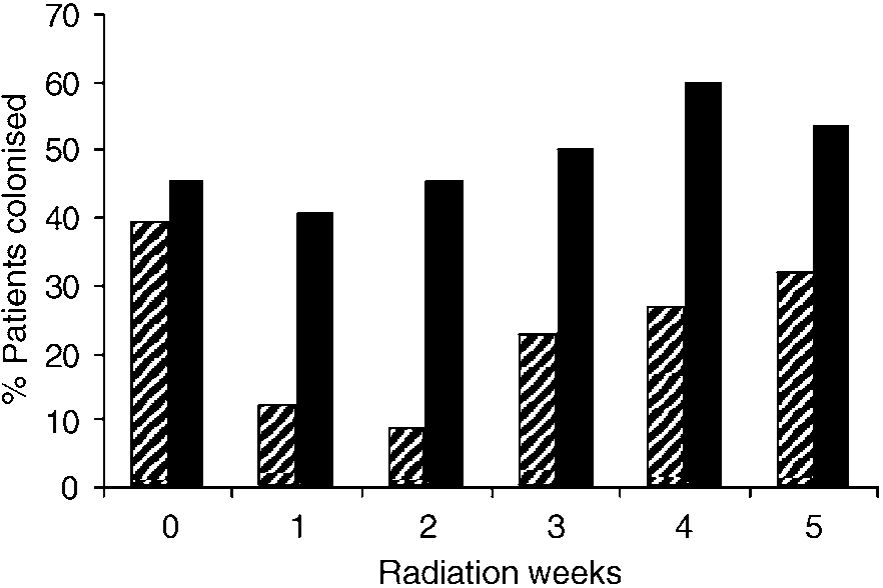
Percentage of patients colonised for *Candida* species for the PTA group (lines) and the placebo group (solid).

The colonisation and carriage of *Candida* species at baseline was equal in both groups (*P*>0.8). During the first two radiation weeks the colonisation for *Candida* species showed an increase in the placebo group and a decrease in the PTA group. After 2 weeks, an increase in both groups was found but the difference between the two groups remained significant during the total study period (*P*<0.05) (Figure 2

).

During the first 4 weeks a significant difference was found for the carriage of *Candida* species (*P*<0.03).

**Figure 3:**
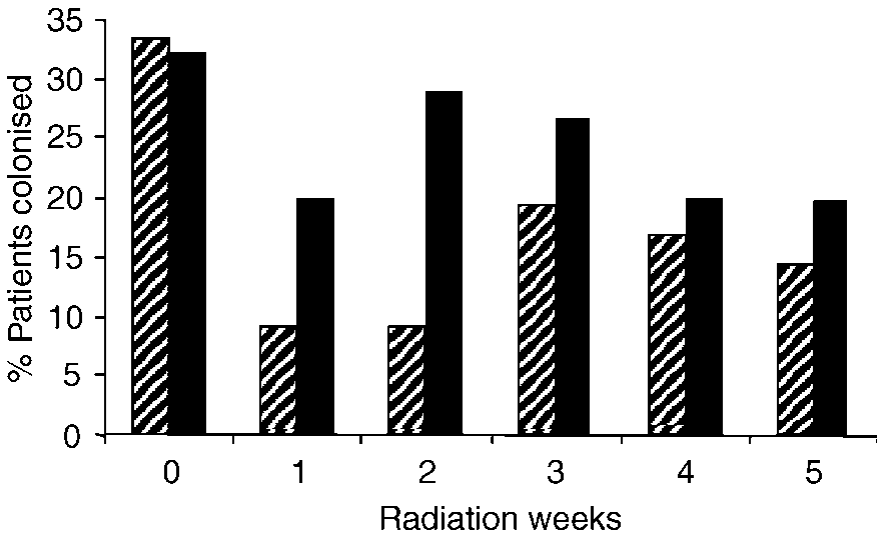
Percentage of patients colonised for aerobic Gram-negative bacilli for the PTA group (lines) and the placebo group (solid).

The colonisation and carriage of aerobic Gram-negative bacilli at baseline was equal in both groups (*P*=0.9). During the radiation period the colonisation in the PTA group was less than in the placebo group, but the difference was only significant in the second week of radiation (*P*=0.05) (Figure 3

).

During the first 2 weeks the carriage of aerobic Gram-negative bacilli was reduced in the PTA group (*P*<0.04). In weeks 3–5 the difference was no longer significant.

All results are summarised in Table 2

**Table 2:** Results of the PTA–placebo group for mean mucositis, weight loss, carriage and colonisation index of *Candida* species and aerobic Gram-negative bacilli

	**0**	**1**	**2**	**3**	**4**	**5**						
**Week**	**X**	**s.d.**	**X**	**s.d.**	**X**	**s.d.**	**X**	**s.d.**	**X**	**s.d.**	**X**	**s.d.**
*Mean mucositis*												
PTA	0	0	0.5	0.7	2.2	1.7	4.6	2.7	4.5	2.7	5.0	2.3
Placebo	0	0	0.3	0.4	2.6	2.0	4.8	2.3	4.9	2.5	5.2	2.8
												
*Weight loss*												
PTA	0	0	−0.3	1.0	0.4	1.3	0.6	2.1	1.0	2.7	1.3	3.0
Placebo	0	0	0.3	0.9^a^	0.6	1.2	1.3	1.7	2.2	2.3	2.8	2.9^a^
												
*Candida col. Index*												
PTA	1.0	1.3	0.4	0.8	0.5	0.9	0.6	0.9	0.8	1.5	1.0	1.5
Placebo	1.1	1.3	1.2	1.6^a^	1.6	1.7^a^	1.7	1.7^a^	2.0	1.7^a^	1.9	1.8^a^
												
*Candida carriage*												
PTA (%)	48		23.5		17.6		31.8		30.8		36	
Placebo (%)	52		76.5^b^		82.4^b^		68.2^b^		69.2^b^		64	
												
*Aerobic Gram-negative bac. col. Index*												
PTA	0.7	1.2	0.4	0.8	0.4	0.9	0.6	1.2	0.5	1.0	0.4	0.9
Placebo	0.7	1.4	0.8	1.1	0.9	1.1^a^	0.8	1.0	0.8	1.4	0.8	1.5
												
*Aerobic Gram-negative bac. Carriage*												
PTA (%)	52.4		23.1		25		42.9		54.5		40	
Placebo (%)	47.6		76.9^b^		75^b^		57.1		45.5		60	

a Represents a significant difference between the PTA and placebo group using an independent sample *t*-test.

b Represents a significant difference between the PTA and placebo group using *χ*^2^ test.

.

## DISCUSSION

In this study, no effect of selective oral flora elimination on mucositis was observed. The development of mucositis follows the same pattern as reported in an earlier cohort study (Spijkervet *et al*, 1989c). According to the WHO score, 80% of the patients in the PTA group and 90% in the placebo group developed mucositis grades 3 and 4. This severity of mucositis is in accordance with the outcomes of Okuno *et al* (1997). In other studies, different outcomes are reported. A significant reduction of mucositis in the PTA group was found by two groups (Spijkervet *et al*, 1990; Kaanders *et al*, 1995). Both studies are nonrandomised clinical trials and the PTA group is compared with a historical control group.

A reduction in mucositis distribution and affected area, dysphagia and weight loss in the PTA group is reported by Symonds *et al*. (1996). From a total of 221 patients in that study, 98 (44%) patients had a larynx carcinoma (PTA=57, placebo=41). Of these patients, the radiation field included only a minor part of the oral mucosa, in which mucositis could develop. In the study of Okuno *et al* only a subjective patient-reported amelioration of mucositis was reported but no reduction was found in clinically observed mucositis (Okuno *et al*, 1997). The PTA group (*n*=54) in that study consisted of an unblinded (*n*=29) and a blinded (*n*=26) group. Only in the unblinded PTA group the mean mucositis, reported by the patients was lower than the placebo group. Recently, it was shown in a randomised study including 77 patients that selective oral flora elimination does not reduce radiation mucositis (Wijers *et al*, 2001). A problem in that study is the short evaluation time of only the first 3 weeks of radiotherapy. Whereas normally development of severe mucositis starts after 3 weeks of radiation (Kaanders *et al*, 1999).

A complicating factor in comparing outcomes from different studies is the assessment method of mucositis. All studies used different scoring methods. Therefore, two scoring methods were used in the current study. The WHO score is a widely accepted method, but this score is a combination of local mucositis signs and general complaints (Anonymous, 1979). The other scoring method in the current study is based only on mucosal signs of mucositis (Spijkervet *et al*, 1989b). It therefore provides a more precise estimation of the mucositis development at the mucosal level. It further makes a comparison possible with outcomes of earlier publications (Spijkervet *et al*, 1989b; Parulekar *et al*, 1998). For future studies, we recommend the use of the OMAS-score from the mucositis study group because this scoring method is a reliable, well-validated and widely accepted method (Sonis *et al*, 1999). This scoring method was published later than the start of the current study and was therefore not used as a scoring method in this study.

In the current study, patients who received PTA lozenges had less weight loss than patients receiving placebo lozenges, assuming a better feeding status of the PTA-group patients (mean difference 1.5 kg). Owing to the minimal effect of PTA on the mucositis level, we found the feeding outcome of minor clinical relevance.

In our study, carriage and colonisation of aerobic Gram-negative bacilli and *Candida* species decreased in the PTA group but was not totally eradicated. These findings are in line with the findings of other studies (Spijkervet *et al*, 1990; Kaanders *et al*, 1995; Symonds *et al*, 1996; Wijers *et al*, 2001). Based on these findings and the development and severity of mucositis it can be concluded that the presence of *Candida* species and aerobic Gram-negative bacilli has no influence on the development of radiation-induced mucositis. The increase of the carriage and colonisation of *Candida* species and aerobic Gram-negative bacilli after 3 weeks of radiation may be explained by the development of xerostomia, which makes dissolving of the lozenges more difficult. Wijers *et al* tried to overcome this problem by using a paste instead of a lozenge. However, the paste appeared to be an unsuccessful form of application because already after randomisation 32% of the patients refused further participation and 77% of the patients dropped out after four study weeks because of bad taste and unpleasant sensation of the paste texture in the mouth (Wijers *et al*, 2001).

In conclusion, PTA lozenges have a positive effect on the quality of feeding and the amount of weight loss but cannot prevent severe mucositis. The presence of *Candida* species and aerobic Gram-negative bacilli has no effect on the development and severity of radiation-induced mucositis. Based on our findings of this randomised clinical trial, we do not recommend this type of supportive care for the reduction or prevention of radiation mucositis.
